# An Electronic Health Record Algorithm’s Performance to Identify Cognitive Impairment in Primary Care

**DOI:** 10.1016/j.jamda.2025.105949

**Published:** 2025-11-12

**Authors:** Christine E. Kistler, Molly E. Lynch, Feng-Chang Lin, Yumei Yang, Winfred Frazier, Laura C. Hanson

**Affiliations:** a Division of Geriatric Medicine, School of Medicine, University of Pittsburgh, Pittsburgh, PA, USA; b Department of Family Medicine, University of North Carolina at Chapel Hill, Chapel Hill, NC, USA; c Cecil G. Sheps Center for Health Services Research, Chapel Hill, NC, USA; d Department of Biostatistics, Gillings School of Global Public Health, Chapel Hill, NC, USA; e Department of Family and Community Medicine, University of Pittsburgh, Pittsburgh, PA, USA; f Division of Geriatric Medicine, School of Medicine, University of North Carolina at Chapel Hill, Chapel Hill, NC, USA; g Palliative Care & Hospice Program, University of North Carolina at Chapel Hill, Chapel Hill, NC, USA

**Keywords:** Cognitive impairment, primary care, Alzheimer’s disease and related dementias

## Abstract

**Objectives:**

Most people with dementia are diagnosed and cared for in primary care. We examined the performance of an electronic health record (EHR) algorithm to identify older adults with dementia or mild cognitive impairment in primary care.

**Design:**

Retrospective cohort study.

**Setting and Participants:**

We identified all adults aged 65+ seen for a routine visit from December 2021 to December 2022 in 59 primary care clinics. We used representative sampling to create a cohort of 525 older adults.

**Methods:**

We used trained research staff to confirm the presence of dementia or mild cognitive impairment via gold standard chart review. We then applied a list of 114 cognitive impairment—related Internaitonal Classification of Diseases, 10^th^ Revision (ICD-10) codes and prescribed dementia medications to the cohort and evaluated the algorithm’s performance characteristics.

**Results:**

Of the representative cohort (n = 525), 59% were women and 81% were White, with an average age of 74.5 ± 6.9 years. Dementia or mild cognitive impairment was present on EHR review in 8.6% (n = 45) of the cohort. The algorithm had a specificity of 98.1%, a sensitivity of 60.0%, a positive predictive value of 0.75, and a negative predictive value of 0.96. The overall predictive performance (F-1 score) was 0.667. Nonspecific ICD-10 was the most common, with “unspecified dementia” representing 17 of 34 ICD-10 codes found by the algorithm.

**Conclusions and Implications:**

Optimization of an EHR algorithm to detect cognitive impairment by combining cognitive impairment—related ICD-10 codes and dementia medications identified people with dementia or mild cognitive impairment in primary care with high specificity and acceptable sensitivity, although ICD-10 codes remained nonspecific.

Nearly 7 million US older adults live with Alzheimer’s disease or a related dementia (ADRD).^1^ This number will grow over the coming decades, with a million new cases added each year by 2060.^2^ Most people with dementia are diagnosed and cared for in primary care.^3^ Because ADRD is a progressive neurodegenerative process, older adults may live more than a decade with the condition and early stages may not be readily recognized.^4,5^ ADRD is chronically under-diagnosed in the United States.^6^ Identifying older adults with ADRD in primary care may improve their quality of life, social support, and advance care planning.^7,8^

Although current evidence does not suggest a benefit of screening for ADRD,^9^ significant efforts are under way in the United States to improve the early detection of ADRD.^10^ Potential benefits to early identification of dementia include access to disease-modifying medications, better management of symptoms and chronic medical conditions, and increased opportunity for advance care planning.^7,11^ Algorithms exist for early ADRD detection but current ones often use natural language processing (NLP) or other data elements that are challenging to reproduce.^12–15^ A pragmatic, low-cost electronic health record (EHR) algorithm, the EHR Risk of Alzheimer’s and Dementia Assessment Rule (eRADAR), is currently being tested in the United States as a means to improve detection of ADRD in an integrated health care system.^16,17^ At this time, no clearly superior or easily reproducible method exists for ADRD detection in primary care.

We have previously developed an EHR algorithm for the detection of hospitalized older adults with moderately severe to late-stage dementia, that is, Global Deterioration Scale stage 5 or later.^12,18,19^ As part of a clinical trial to improve advance care planning for people with dementia or mild cognitive impairment in primary care, we expanded the list of International Classification of Diseases, 10^th^ Revision (ICD-10) codes and examined the addition of other cognitive impairment—related factors readily found in the EHR. Our hypothesis is that an ICD-10 algorithm would have adequate performance characteristics to use in a clinical trial. This study evaluated the performance of an EHR algorithm to identify older adults across the spectrum of dementia in primary care practices in a large public health care system.

## Methods

### Study Design

We used a clinical data warehouse (CDW) from a single large health care network in North Carolina to develop a cohort of all adults aged 65 and older seen for a routine primary care visit from December 14, 2021, to December 14, 2022, in 59 affiliated primary care clinics. The data warehouse contains all clinical encounters within the health care network. We randomly selected 10,000 older adults with eligible clinic visits and used a proportional sampling process to create a demographically matched cohort of 525 patients, using PROC SURVESELECT in SAS. This study was approved by the institutional review board at the University of North Carolina at Chapel Hill (IRB #22-3230).

We adapted an existing EHR algorithm to identify hospitalized patients with late-stage dementia by expanding the list of ICD-10 codes to include any cognitive impairment.^18^ The code-based algorithm included the presence of any 114 related ICD-10 codes (Supplementary Material). We then examined similar published algorithms to identify other factors that improve case-finding older adults with cognitive impairment.^13,14,16^ We included any factors easily available from the CDW to determine if they could improve the algorithm’s performance characteristics. Our trained research coordinators with cognitive impairment—related expertise then conducted structured chart reviews to determine the presence of any cognitive impairment, including all stages of cognitive impairment. The research coordinators were trained by geriatricians with expertise in dementia diagnosis (C.E.K. and L.C.H.) and worked closely with these clinicians to adjudicate discrepancies or understanding in the chart review process. We then compared the algorithm with the structured chart reviews.

### Population

To facilitate chart reviews, we used the demographic proportions from the 10,000 randomly selected older adults to sample a smaller population of 525 participants. We proportionally matched participants on age, gender, race, comorbidity, and insurance status. We excluded primary care clinic visits in which patients did not receive routine care from a physician or advanced practice provider (ie, laboratory or nursing visits, or for appointments in urgent care).

### Measures

The primary outcome of the study was the rate of cognitive impairment including all stages of cognitive impairment in the cohort via an automated data abstraction of 114 related ICD-10 codes from the CDW and an expert chart review from research coordinators with extensive experience in dementia ascertainment.^12,18^ Additional factors abstracted to enhance the algorithm included dementia-related medications, speech therapy visits for communication and/or cognitive assessment or treatment, or 1 or more clinic “no shows,” which might be signs of mild cognitive impairment before a formal ICD-10 code is placed in the medical record.

Given concerns that the diagnosis of dementia or mild cognitive impairment might be biased by demographic characteristics, we also abstracted key demographic characteristics such as race, ethnicity, and gender. We also examined other clinically relevant characteristics such as comorbidity, rurality, and socioeconomic disadvantage. Comorbidity was measured via the Charlson comorbidity index (CCI), a highly validated and widely used measure of comorbidity.^20^ The CCI includes 19 common medical conditions and an increasing score indicates increasing comorbidity. We measured rurality via the Rural-Urban Continuum Codes (RUCC) for each of the clinics, as patient-level data were not available.^21^ A RUCC ranges from 1 to 9 with codes 1 to 3 being associated with metropolitan/urban-sized categories and 4 to 9 being nonmetro/rural-sized categories. We measured socioeconomic disadvantage through the area deprivation index. The area deprivation index is a ranked metric of neighborhood socioeconomic disadvantage, with 1 being the least disadvantaged and 100 being the most disadvantaged neighborhoods nationwide.^22^

### Analysis

We used descriptive statistics to compare the overall randomly selected patient cohort (N = 10,000) to the proportionally matched sample (n = 525). We examined the performance characteristics of the ICD-10 code algorithm to the cohort to identify older adults with probable cognitive impairment as compared with the chart review gold standard. We added each cognitive impairment—related factor separately and combined to determine the additional benefit of these factors in improving the algorithm’s performance characteristics. We determined their sensitivity/specificity, negative/positive predictive values, and overall predictive performance (via an F-1 score). Last, we examined the rates of cognitive impairment for disparities in diagnosis by demographic characteristics such as race, gender, insurance status, and area deprivation index (ADI) for their clinic location using a linear regression model.^23^

## Results

Of the representative cohort (n = 525), 59% were women, 81% were White, and almost all had Medicare (93%) with an average age of 74.5 ± 6.9 years (Table 1). The most common medical condition was diabetes (20%), followed by chronic kidney disease and chronic obstructive pulmonary disease (both 14%). The average RUCC score was 2.3 ± 1.4, indicating most participants were affiliated with a large metropolitan area. The average CCI was 1.7 ± 2.2, indicating 2 of every 3 participants had at least 2 chronic medical conditions. The average ADI score of the clinic was 46 ± 21.5, which indicates average levels of deprivation.

### Performance Characteristics of EHR Algorithms

EHR review found cognitive impairment in 8.6% (n = 45) of the cohort, with most having either other symptoms/signs of impaired cognitive function (n = 16) or unspecified dementia (n = 10). Extrapolating the results from the proportionally matched cohort to the larger 10,000-person cohort, we would expect a similar 8.6% would have cognitive impairment, or 860 individuals.

Only the addition of dementia-related medications improved the performance of the ICD-10 code—based algorithm (Table 2). The algorithm, which included the addition of dementia medications, had a specificity of 98.1% and a sensitivity of 60.0%, with a positive predictive value of 0.75 and a negative predictive value of 0.96. Of the 36 older adults identified by the algorithm as having cognitive impairment, 27 of them (75%) were confirmed to have impairment. The overall predictive performance (F-1 score) that examines the global performance of the algorithm was 0.667. Other factors, such as the presence of “no shows,” the receipt of speech language therapy, antidepressants, and so foth, contributed minimally to the algorithm’s performance and were not included. The algorithm performed equally well for subpopulations and no demographic characteristics were associated with disparities in the rates of detection of mild cognitive impairment or dementia.

We examined the ICD-10 codes that were triggered to identify people with dementia or mild cognitive impairment by the algorithm to see if any patterns emerged (Table 3). The most common ICD-10 code type found by either the algorithm or the chart review was “unspecified dementia” in 17 cases. The “cerebrovascular disease” codes were the next most common (8 of 34) followed by “dementia in other diseases” (4 of 34). Similar patterns were found during chart review, although “degeneration of the nervous system, various causes” was second most common (6 of 29), and “age-related cognitive decline” was also present (3 of 29). The algorithm found 12 instances of cognitive impairment due to dementia-related medications alone.

## Discussion

Among a cohort of older adults, the addition of dementia medications to cognitive impairment—related ICD-10 codes resulted in an algorithm with good performance characteristics. The algorithm will be used to identify a cohort of people with dementia or mild cognitive impairment to test whether an intervention in primary care improves advance care planning (ACP) and goals of care, similar to our previous work.^24^ No disparities in ADRD diagnosis were found by race or clinic’s ADI. Given the algorithm’s high specificity, most individuals identified by the algorithm with cognitive impairment had chart review—confirmed dementia or mild cognitive impairment, which is useful as part of a clinical trial. However, the low sensitivity means that additional efforts, like eRADAR, may be needed to locate those people with dementia that this study’s algorithm falsely misses. The addition of dementia medications improved algorithm sensitivity, although the algorithm missed 18 individuals found on chart review.

Different algorithms to detect people with dementia may serve different purposes. The algorithm that served as the basis for this study’s algorithm is currently being used in a multisite trial in the inpatient setting as part of a palliative care intervention.^25^ This algorithm was designed to target those with moderately severe dementia or worse. Another trial is using eRADAR to detect patients with unrecognized dementia for further interventions in primary care.^16,17^ We could not locate a systematic review of EHR algorithms for detecting dementia or mild cognitive impairment. However, a recent Cochrane review of primary care providers’ diagnosis of dementia or cognitive impairment found performance characteristics similar to our algorithm, which makes sense given our algorithm relies on providers using ICD-10 codes.^26^ This algorithm will be used to identify a cohort of people with dementia or mild cognitive impairment in primary care to assess the receipt of ACP and goals of care conversations, as well as health care utilization, in a randomized controlled trial based on a prior pre-post observational study.^24^

Knowledge about the performance of these algorithms in primary care can inform efforts to provide people with dementia further supports, such as with the Guiding an Improved Dementia Experience (GUIDE) Model demonstration recently launched by the Centers for Medicare and Medicaid Services.^27^ Upward of 80% of people with dementia become homebound at some point in their disease trajectory.^28^ These people will struggle to access their primary care teams and care becomes increasingly fragmented.^29^ ACP and identifying the goals of care of people with dementia can help ensure that they receive the kind of care that matches their preferences across the spectrum of disease.

Last, we found people with dementia or mild cognitive impairment continue to have vague and nonspecific cognitive impairment—related diagnoses in their EHR, lacking disease severity. A recent scoping review found pervasive underdiagnosis of dementia particularly in early stages.^4^ We found that gold standard chart review identified other codes possibly associated with cognitive impairment. These codes for “Memory Difficulties” or “Other symptoms and signs involving cognitive functions and awareness” are also vague. Including these codes in the algorithm would have potentially improved the overall performance of the algorithm to 0.682, increasing sensitivity to 64.4% and reducing the specificity to 97.7%. Additional codes may improve the performance of the algorithm and warrant further study in other primary care populations. Interestingly, prior work found that inclusion of dementia-related medications did not improve the performance characteristics of an automated algorithm in EPIC that used ICD codes, medications, and NLP logic.^13^ Last, further efforts to encourage the use of dementia ICD-10 codes in clinical care may help improve the ability for large health systems or insurance providers to identify people with dementia or mild cognitive impairment for targeted outreach and support. Based on our findings and others, the diagnosis of dementia remains challenging and may need further clinical support to fully capture people with dementia or mild cognitive impairment.

This study is limited by the use of the structured data elements in the data warehouse. Other work to combine ICD diagnoses and artificial intelligence including NLP logic may further improve the performance characteristics of the current algorithm.^13–15^ The addition of NLP appears promising, but is not easily transportable across health care systems, making the dissemination of such an algorithm even more challenging. We also did not include other factors that might change the performance of the algorithm such as laboratory results for dementia-related biomarkers, delirium-related hospitalizations, or other classes of medications given potential overlap with the ICD-10 codes or dementia-related medications. In addition, we did not review all 10,000 charts for the presence of cognitive impairment. The true performance characteristics may differ from our current findings, and the algorithm should be tested in other primary care systems, which should be relatively easy given the discrete structured data elements used. Last, we did not perform confirmatory testing on the identified people with dementia or mild cognitive impairment to determine clinical accuracy, as this algorithm is currently developed for research purposes. Further work will be needed before the algorithm is ready for clinical care, and an algorithm that works for the very heterogeneous population in primary care may not work in neurology or specialties.

## Conclusions

Optimization of an EHR algorithm to detect cognitive impairment by combining cognitive impairment—related ICD-10 codes and dementia medications identified people with dementia in primary care with high specificity and acceptable sensitivity, although ICD-10 codes remained nonspecific. Further work is needed to optimize identifying people with dementia or mild cognitive impairment in primary care and may involve the addition of NLP logic. The field would benefit from a systematic review of EHR and NLP algorithms. Algorithms in primary care have the potential to facilitate access to dementia-specific services and supports.

## Supplementary Material

1

Supplementary data related to this article can be found online at https://doi.org/10.1016/j.jamda.2025.105949.

## Figures and Tables

**Table 1:** Participant Characteristics, n (%) or mean (SD)

Characteristics	Sampled Patients n = 525	Overall Population N = 10,000	*P* Value
Female	312 (59.4)	5941 (59.4)	1.00
Age	74.5 (6.89)	74.4 (6.84)	.942
Race			1.00
White	423 (80.6)	8060 (80.6)	—
African American	79 (15.0)	1501 (15.0)	—
Other	21 (4.0)	396 (4.0)	—
Hispanic or Latino	9 (1.7)	185 (1.9)	.972
ADI national score of Primary Care Clinic, mean (SD)	46 (21.5)		
RUCC, mean (SD)	2.3 (1.4)		
CCI, mean (SD)	1.7 (2.2)		
Diabetes (without complications)	105 (20.0)		
Moderate or severe renal disease (chronic kidney disease)	73 (13.9)		
Chronic obstructive pulmonary disease	73 (13.9)		
Solid tumor (localized or nonmetastatic)	62 (11.8)		
Peripheral vascular disease	58 (11.0)		
Type of insurance			.603
Medicare	490 (93.4)	9346 (93.5)	
Other	29 (5.5)	593 (5.9)	
None	6 (1.1)	61 (0.6)	—
Clinic-level characteristic			
Number of clinics	59	—	—
Number of primary care providers	3.69 (4.88)	—	—

Participants represent those seen by 261 unique providers.

**Table 2:** Accuracy of Cognitive Impairment Diagnosis Including Sensitivity (TP) and Specificity (TN), n = 525

Algorithm*- F1 Score = 0.667	Chart Review Cognitive Impairment Status			
	Present	Not Present	Total	Predictive Values (PV)
Present	**TP = 27 (60.0%)**	FP = 9 (1.9%)	36	Positive PV = 0.75
Not present	FN = 18 (40.0%)	**TN = 469 (98.1%)**	487	Negative PV = 0.96
Total	45	478	523	

FN, false negative; FP, false positive; TN, true negative; TP, true positive.

Bolded frequencies indicate correct decision.

* Includes dementia medications.

**Table 3:** ADRD Structured Data Element Categories, N = 525

ADRD Structured Data Elements	Algorithm, n (%)	Chart Review, n (%)	*P* Value
Unspecified dementia			
Severity not listed	17 (3.2)	10 (1.9)	.24
Mild	1 (0.2)	0	1.00
Moderate	—	—	
Severe	—	—	
Cerebrovascular disease	8 (1.5)	0	.01
Degeneration of nervous system, various causes	0	6 (1.1)	.03
Alzheimer’s disease and other frontotemporal dementia	3 (0.6)	3 (0.6)	1.00
Vascular dementia	1 (0.2)	3 (0.6)	.62
Dementia in other diseases	4 (0.8)	3 (0.6)	1.00
Age-related cognitive decline	0	3 (0.6)	.25
Mental disorders due to known physiological conditions	0	1 (0.2)	1.00
Alcohol or other substance use	0	0	1.00
Creutzfeldt-Jakob disease, unspecified	0	0	1.00
Other ICD-10—related data element	0	9 (1.7)*	.004
No ICD-10—related data element present	12 (2.2)^†^	8 (1.5)^‡^	.50
Total number of data elements^§^	46	50	

* R41.3 Memory Difficulties: 8, R41.89 Other symptoms and signs involving cognitive functions and awareness: 1.

† These are due to the dementia-related medications.

‡ These data elements come from the experience of the expert chart review.

§ Total number of data elements identified via the algorithm and chart review are greater than the n = 36 total cases, modified algorithm or the n = 45 total cases, chart review due to individuals having more than one medication or ICD-10 data element.
