# Supplementary material for: An Electronic Health Record Algorithm’s Performance to Identify Cognitive Impairment in Primary Care
Source: J Am Med Dir Assoc. Author manuscript; Available in PMC 2026 Jun 23. (PMC13290007; doi:10.1016/j.jamda.2025.105949)
Supplement: 1 [file NIHMS2183351-supplement-1.docx]

| **DIAGNOSIS CODE (ICD 10)** | **Name/Description** |
| --- | --- |
| A81.00 | Creutzfeldt-Jakob disease, unspecified |
| F01 | Vascular dementia |
| F01.5 | Vascular dementia |
| F01.50 | Vascular dementia, unspecified severity, without behavioral disturbance, psychotic disturbance, mood disturbance, and anxiety |
| F01.51 | Vascular dementia, unspecified severity, with behavioral disturbance |
| F01.511 | Vascular dementia, unspecified severity, with agitation |
| F01.518 | Vascular dementia, unspecified severity, with other behavioral disturbance |
| F01.52 | Vascular dementia, unspecified severity, with psychotic disturbance |
| F01.53 | Vascular dementia, unspecified severity, with mood disturbance |
| F01.54 | Vascular dementia, unspecified severity, with anxiety |
| F01.A0 | Vascular dementia, mild, without behavioral disturbance, psychotic disturbance, mood disturbance, and anxiety |
| F01.A11 | Vascular dementia, mild, with agitation |
| F01.A18 | Vascular dementia, mild, with other behavioral disturbance |
| F01.A2 | Vascular dementia, mild, with psychotic disturbance |
| F01.A3 | Vascular dementia, mild, with mood disturbance |
| F01.A4 | Vascular dementia, mild, with anxiety |
| F01.B0 | Vascular dementia, moderate, without behavioral disturbance, psychotic disturbance, mood disturbance, and anxiety |
| F01.B11 | Vascular dementia, moderate, with agitation |
| F01.B18 | Vascular dementia, moderate, with other behavioral disturbance |
| F01.B2 | Vascular dementia, moderate, with psychotic disturbance |
| F01.B3 | Vascular dementia, moderate, with mood disturbance |
| F01.B4 | Vascular dementia, moderate, with anxiety |
| F01.C0 | Vascular dementia, severe, without behavioral disturbance, psychotic disturbance, mood disturbance, and anxiety |
| F01.C11 | Vascular dementia, severe, with agitation |
| F01.C18 | Vascular dementia, severe, with other behavioral disturbance |
| F01.C2 | Vascular dementia, severe, with psychotic disturbance |
| F01.C3 | Vascular dementia, severe, with mood disturbance |
| F01.C4 | Vascular dementia, severe, with anxiety |
| F02 | Dementia in other diseases classified elsewhere |
| F02.2 | Dementia in Huntington’s Disease |
| F02.4 | Dementia in human immunodeficiency virus (HIV) disease |
| F02.8 | Dementia in other diseases classified elsewhere |
| F02.80 | Dementia in other diseases classified elsewhere, unspecified severity, without behavioral disturbance, psychotic disturbance, mood disturbance, and anxiety |
| F02.81 | Dementia in other diseases classified elsewhere, unspecified severity, with behavioral disturbance |
| F02.811 | Dementia in other diseases classified elsewhere, unspecified severity, with agitation |
| F02.818 | Dementia in other diseases classified elsewhere, unspecified severity, with other behavioral disturbance |
| F02.82 | Dementia in other diseases classified elsewhere, unspecified severity, with psychotic disturbance |
| F02.83 | Dementia in other diseases classified elsewhere, unspecified severity, with mood disturbance |
| F02.84 | Dementia in other diseases classified elsewhere, unspecified severity, with anxiety |
| F02.A0 | Dementia in other diseases classified elsewhere, mild, without behavioral disturbance, psychotic disturbance, mood disturbance, and anxiety |
| F02.A11 | Dementia in other diseases classified elsewhere, mild, with agitation |
| F02.A18 | Dementia in other diseases classified elsewhere, mild, with other behavioral disturbance |
| F02.A2 | Dementia in other diseases classified elsewhere, mild, with psychotic disturbance |
| F02.A3 | Dementia in other diseases classified elsewhere, mild, with mood disturbance |
| F02.A4 | Dementia in other diseases classified elsewhere, mild, with anxiety |
| F02.B0 | Dementia in other diseases classified elsewhere, moderate, without behavioral disturbance, psychotic disturbance, mood disturbance, and anxiety |
| F02.B11 | Dementia in other diseases classified elsewhere, moderate, with agitation |
| F02.B18 | Dementia in other diseases classified elsewhere, moderate, with other behavioral disturbance |
| F02.B2 | Dementia in other diseases classified elsewhere, moderate, with psychotic disturbance |
| F02.B3 | Dementia in other diseases classified elsewhere, moderate, with mood disturbance |
| F02.B4 | Dementia in other diseases classified elsewhere, moderate, with anxiety |
| F02.C0 | Dementia in other diseases classified elsewhere, severe, without behavioral disturbance, psychotic disturbance, mood disturbance, and anxiety |
| F02.C11 | Dementia in other diseases classified elsewhere, severe, with agitation |
| F02.C18 | Dementia in other diseases classified elsewhere, severe, with other behavioral disturbance |
| F02.C2 | Dementia in other diseases classified elsewhere, severe, with psychotic disturbance |
| F02.C3 | Dementia in other diseases classified elsewhere, severe, with mood disturbance |
| F02.C4 | Dementia in other diseases classified elsewhere, severe, with anxiety |
| F03 | Unspecified dementia |
| F03.9 | Unspecified dementia |
| F03.90 | Unspecified dementia, unspecified severity, without behavioral disturbance, psychotic disturbance, mood disturbance, and anxiety |
| F03.91 | Unspecified dementia, unspecified severity, with behavioral disturbance |
| F03.911 | Unspecified dementia, unspecified severity, with agitation |
| F03.918 | Unspecified dementia, unspecified severity, with other behavioral disturbance |
| F03.92 | Unspecified dementia, unspecified severity, with psychotic disturbance |
| F03.93 | Unspecified dementia, unspecified severity, with mood disturbance |
| F03.94 | Unspecified dementia, unspecified severity, with anxiety |
| F03.A0 | Unspecified dementia, mild, without behavioral disturbance, psychotic disturbance, mood disturbance, and anxiety |
| F03.A11 | Unspecified dementia, mild, with agitation |
| F03.A18 | Unspecified dementia, mild, with other behavioral disturbance |
| F03.A2 | Unspecified dementia, mild, with psychotic disturbance |
| F03.A3 | Unspecified dementia, mild, with mood disturbance |
| F03.A4 | Unspecified dementia, mild, with anxiety |
| F03.B0 | Unspecified dementia, moderate, without behavioral disturbance, psychotic disturbance, mood disturbance, and anxiety |
| F03.B11 | Unspecified dementia, moderate, with agitation |
| F03.B18 | Unspecified dementia, moderate, with other behavioral disturbance |
| F03.B2 | Unspecified dementia, moderate, with psychotic disturbance |
| F03.B3 | Unspecified dementia, moderate, with mood disturbance |
| F03.B4 | Unspecified dementia, moderate, with anxiety |
| F03.C0 | Unspecified dementia, severe, without behavioral disturbance, psychotic disturbance, mood disturbance, and anxiety |
| F03.C11 | Unspecified dementia, severe, with agitation |
| F03.C18 | Unspecified dementia, severe, with other behavioral disturbance |
| F03.C2 | Unspecified dementia, severe, with psychotic disturbance |
| F03.C3 | Unspecified dementia, severe, with mood disturbance |
| F03.C4 | Unspecified dementia, severe, with anxiety |
| F04 | Amnestic disorder due to known physiological condition |
| F05 | Delirium due to known physiological condition |
| F06.0 | Psychotic disorder with hallucinations due to known physiological condition |
| F06.8 | Other specified mental disorders due to known physiological condition |
| F10.231 | Alcohol dependence with withdrawal delirium |
| F10.27 | Alcohol dependence with alcohol-induced persisting dementia |
| F10.96 | Alcohol use, unspecified with alcohol-induced persisting amnestic disorder |
| F10.97 | Alcohol use, unspecified with alcohol-induced persisting dementia |
| F19.97 | Other psychoactive substance use, unspecified with psychoactive substance-induced persisting dementia |
| F99 | Mental disorder, not otherwise specified |
| G10 | Huntington's disease |
| G30.0 | Alzheimer's disease with early onset |
| G30.1 | Alzheimer's disease with late onset |
| G30.8 | Other Alzheimer's disease |
| G30.9 | Alzheimer's disease, unspecified |
| G31.01 | Pick's disease |
| G31.09 | Other frontotemporal dementia |
| G31.1 | Senile degeneration of brain, not elsewhere classified |
| G31.2 | Degeneration of nervous system due to alcohol |
| G31.83 | Dementia with Lewy bodies |
| G31.84 | Mild cognitive impairment, so stated |
| G31.89 | Other specified degenerative diseases of nervous system |
| G31.9 | Degenerative disease of nervous system, unspecified |
| G91.4 | Hydrocephalus in diseases classified elsewhere |
| I67.89 | Other cerebrovascular disease |
| I67.9 | Cerebrovascular disease, unspecified |
| I69.31 | Cognitive deficits following cerebral infarction |
| I69.81 | Cognitive deficits following other cerebrovascular disease |
| I69.91 | Cognitive deficits following unspecified cerebrovascular disease |
| R41.81 | Age-related cognitive decline |

| **Dementia-Related Medications** |
| --- |
| Galantamine |
| Donepezil |
| Rivastigmine |
| Memantine |
| Huperzine |
